# Duodenal Indigestion

**Published:** 1893-08-12

**Authors:** 


### EDITOR'S LETTER-BOX.

[Our correspondents are reminded that prolixity is a great bar to publication, and that brevity of style and conciseness of statement greatly facilitate early insertion.]

### DUODENAL INDIGESTION.

"R. M. S." writes: In your issue of July 22nd, Dr. Hanbury Frere, in his article on "A Form of Indigestion and its Treatment," deplores the paucity of literature on the subject of duodenal indigestion or dyspepsia. He is evidently not acquainted with an admirable work on the subject by W. H. Allchin, "The Nature and Causes of Duodenal Indigestion." Bradshaw Lecture, 1891.

